# Na,K-ATPase Acts as a Beta-Amyloid Receptor Triggering Src Kinase Activation

**DOI:** 10.3390/cells11172753

**Published:** 2022-09-03

**Authors:** Irina Yu. Petrushanko, Artem M. Tverskoi, Evgeny P. Barykin, Aleksandra V. Petrovskaya, Maria A. Strelkova, Olga G. Leonova, Anastasia A. Anashkina, Anna P. Tolstova, Alexei A. Adzhubei, Anna Yu. Bogdanova, Alexander A. Makarov, Vladimir A. Mitkevich

**Affiliations:** 1 Engelhardt Institute of Molecular Biology, Russian Academy of Sciences, 119991 Moscow, Russia; 2 Department of Microbiology, Immunology, and Tropical Medicine, Washington University School of Medicine and Health Sciences, Washington, DC 63110-1010, USA; 3 Institute of Veterinary Physiology, Vetsuisse Faculty and the Zurich Center for Integrative Human Physiology (ZIHP), University of Zurich, CH-8057 Zurich, Switzerland

**Keywords:** Na,K-ATPase, beta-amyloid, Src kinase, hypoxia, receptor function

## Abstract

Beta-amyloid (Aβ) has a dual role, both as an important factor in the pathology of Alzheimer’s disease and as a regulator in brain physiology. The inhibitory effect of Aβ_42_ oligomers on Na,K-ATPase contributes to neuronal dysfunction in Alzheimer’s disease. Still, the physiological role of the monomeric form of Aβ_42_ interaction with Na,K-ATPase remains unclear. We report that Na,K-ATPase serves as a receptor for Aβ_42_ monomer, triggering Src kinase activation. The co-localization of Aβ_42_ with α1- and β1-subunits of Na,K-ATPase, and Na,K-ATPase with Src kinase in SH-SY5Y neuroblastoma cells, was observed. Treatment of cells with 100 nM Aβ_42_ causes Src kinase activation, but does not alter Na,K-ATPase transport activity. The interaction of Aβ_42_ with α1β1 Na,K-ATPase isozyme leads to activation of Src kinase associated with the enzyme. Notably, prevention of Na,K-ATPase:Src kinase interaction by a specific inhibitor pNaKtide disrupts the Aβ-induced Src kinase activation. Stimulatory effect of Aβ_42_ on Src kinase was lost under hypoxic conditions, which was similar to the effect of specific Na,K-ATPase ligands, the cardiotonic steroids. Our findings identify Na,K-ATPase as a Aβ_42_ receptor, thus opening a prospect on exploring the physiological and pathological Src kinase activation caused by Aβ_42_ in the nervous system.

## 1. Introduction

Beta-amyloid (Aβ) is an endogenous peptide that plays both physiological and pathophysiological roles in the human brain. On the one hand, toxic Aβ oligomers are involved in the pathogenesis of Alzheimer’s disease (AD), leading to impaired functioning and death of neurons [1,2,3]. On the other hand, Aβ is a regulatory molecule with an important part in the normal functioning of the nervous tissue [4,5]. In particular, the activation of Fyn-kinase from the Src kinase family by Aβ_42_ is required for the maturation of oligodendrocytes [6]. Endogenous Aβ_42_ in the central nervous system is important for synaptic plasticity and memory [7,8].

However, continuous activation of signaling cascades with increasing Aβ_42_ levels may contribute to disruption of cell function in brain tissue. Accordingly, the proinflammatory activation of microglia characteristic of AD occurs due to the Src kinase activation and TREM2-mediated activation of the glycogen synthase kinase-3β affected by Aβ_42_ [9,10]. Concurrent activation of Fyn-kinase in neurons [11] and Src kinase in microglia by Aβ oligomers [12] leads to the synapse loss and behavioral disorders associated with AD [13]. Hence, while preventing pathological cascades, it is important to preserve the physiological effects of Aβ. To specifically suppress the pathological cascades, it is essential to uncover the Aβ receptors and unravel the downstream signaling induced by the interaction of Aβ with those receptors.

One of the candidates that recently emerged as an Aβ receptor is Na,K-ATPase, a membrane transporter providing Na^+^ and K^+^ homeostasis in all animal cells. Its activity is critically important for normal neuronal function. Aβ was shown to interact with the Na,K-ATPase and at supra-physiologically high doses inhibits activity of the enzyme in a time- and dose-dependent manner [14,15]. These pathologically high doses of Aβ can be achieved in the AD brain. A decrease in the activity of Na,K-ATPase under exposure to Aβ_42_ was demonstrated both in vivo [14,16] and in vitro [14,15,17] and occurs as a result of binding of the Aβ to Na,K-ATPase [14,15] and its subsequent aggregation utilizing the first molecule of Aβ_42_ bound to the enzyme as a seed [18]. This inhibitory effect of Aβ_42_ was suggested to be the cause of neuronal dysfunction at the early stages of AD [14,15,16,19].

Despite being toxic in high concentrations and/or aggregated state, monomeric Aβ_42_ is neuroprotective in nanomolar concentrations [20,21]. We hypothesized that the specific interaction of Aβ_42_ monomers with Na,K-ATPase might produce a functional effect at normal physiological concentrations of the peptide. We made our assumption by analogy with the function of cardiotonic steroids (CTSs), the only currently known physiological ligands for Na,K-ATPase in the peripheral tissues. High doses of CTSs inhibit the transport function of Na,K-ATPase, while sub-inhibitory doses of CTSs activate receptor function of the enzyme [22,23,24]. The function of Na,K-ATPase as a CTSs receptor is mainly implemented through Src kinase. In cells, there is a pool of Src kinase associated with Na,K-ATPase, and the binding of CTS to Na,K-ATPase leads to changes in this interaction and the activation of Src [24,25,26,27].

It is known that the treatment of cells with Aβ leads to the activation of signaling cascades through Src kinase family proteins [6,9,13,15,28]. However, the mediator between monomers of Aβ and Src kinase is not known. In this study, we analyzed whether Na,K-ATPase has the role of such a mediator and constitutes as a receptor for Aβ_42_, with their interaction leading to the activation of Src kinase.

## 2. Materials and Methods

### 2.1. Cell Culture

Human neuroblastoma SH-SY5Y cells from the American Type Culture Collection were cultured in RPMI-1640 media (Gibco, ThermoFisher Scientific, Waltham, MA, USA), ThermoFisher Scientific, MA, USA), containing 10% fetal bovine serum (FBS; Gibco, ThermoFisher Scientific, MA, USA), 100 units/mL penicillin, 100 µg/mL streptomycin, sodium pyruvate and glutamax (Gibco, ThermoFisher Scientific, MA, USA) in T-25 and T-75 culture flasks at 37 °C in humid atmosphere with 5% CO_2_ (passages did not exceed 15). For confocal microscopy and in situ proximity ligation assay (PLA) experiments, 35 mm glass-based Petri dishes (Nunc, Rochester, NY, USA, 150680) were used. SH-SY5Y cells were seeded in a quantity of 15,000 per Petri dish. For Western blot, measurements of Na,K-ATPase transport activity, assessment of Na,K-ATPase level and redox status, SH-SY5Y cells were grown in 6- or 12-well plates until 80–90% confluency was achieved.

### 2.2. Aβ_42_ Preparation

Synthetic peptide Aβ_42_:

[H2N]-DAEFRHDSGYEVHHQKLVFFAEDVGSNKGAIIGLMVGGVVIA-[COOH] and reverse peptide 42−1 (CP_Aβ_) was purchased from Biopeptide (San Diego, CA, USA). Preparation of the monomeric form of Aβ_42_ was performed as described elsewhere [15,29]. Cold hexafluoroisopropanol (Fluka) was added to dry Aβ_42_ to a concentration of 1 mM and incubated for 60 min at room temperature. Then, the peptide solution was put on ice for 10 min and aliquoted into non-siliconized microcentrifuge tubes (0.56 mg peptide per tube). Peptide in the tubes was dried under vacuum using Eppendorf Concentrator 5301. Dried peptide was stored at −80 °C. 2.5 mM peptide stock solution was prepared by adding 20 μL of 100% anhydrous DMSO (Sigma-Aldrich, St. Louis, MO, USA) to 0.22 mg peptide and incubating for 1 h at room temperature. The peptide was further diluted to the required concentration with buffer solution. The equivalent amount of DMSO was added to the control samples in all experiments. Only freshly prepared peptide solutions were used for all experiments. As shown by us earlier [15] by dynamic light scattering and turbidity measurements, these Aβ_42_ solutions do not contain particles in the ranges of 0.6–10 nm and 1–100 nm. Monomers constituted 80% in Aβ_42_ preparation [15].

### 2.3. pNaKtide Preparation

Synthetic peptide pNaktide:

[H2N]-GRKKRRQRRRPPQSATWLALSRIAGLCNRAVFQ-[COOH] was purchased from LifeTein (Somerset, NJ, USA) in accordance with the sequence given in [30]. Dry pNaKtide was dissolved in deionized water to a concentration of 1 mM and was stored at −25 °C. The peptide was further diluted to the required concentration with buffer solution or culture media.

### 2.4. Co-Localization Studies with Confocal Microscopy

SH-SY5Y cells grown in 35 mm glass-based Petri dishes were washed with serum-free RPMI medium and then incubated with 40 µM Aβ_42_ for 2 h in a serum-free medium. 

#### 2.4.1. Cell Staining for the β1-Subunit of Na,K-ATPase and Aβ_42_

After incubation with Aβ_42_, cells were washed with a serum-free RPMI medium and stained with polyclonal rabbit antibodies for β-amyloid (dilution in RPMI medium 1:50, Sigma-Aldrich, St. Louis, MO, USA, A3356, epitope—Aβ(25–35)) and monoclonal mouse antibodies for β1-subunit of Na,K-ATPase (dilution in RPMI medium 1:100, Millipore, Burlington, MA, USA, 05-382, epitope—ectodomain of β1-subunit) for an hour in a CO_2_-incubator at 37 °C. After the washout, the cells were stained with secondary anti-rabbit IgG conjugated to Texas Red (Santa Cruz Biotechnology, Dallas, TX, USA, sc-2780) and anti-mouse IgG conjugated to fluorescein isothiocyanate (FITC; Santa Cruz Biotechnology, Dallas, TX, USA, sc-2010) for 30 min at 37 °C in CO_2_-incubator. In the cells without Aβ_42_ treatment amyloid staining was not observed (Supplementary Figure S1). In the cells not treated with primary antibodies (secondary antibodies only) fluorescent signal was absent (Supplementary Figure S1).

#### 2.4.2. Cell Staining for the α1-Subunit of Na,K-ATPase and Aβ_42_

After incubation with Aβ_42_, the cells were washed with serum-free RPMI medium and stained with antibodies for Aβ_42_ (dilution 1:50 in RPMI medium, Sigma-Aldrich, St. Louis, MO, USA, A3356) for an hour in a CO_2_-incubator at 37 °C. Then, the cells were washed with PBS and fixed with 4% paraformaldehyde for 10 min at room temperature. Next, the cells were washed with PBS and permeabilized with 0.5% saponin in PBS for 10 min at room temperature. Further, the monoclonal mouse antibodies were used to stain the α1-subunit of Na,K-ATPase (dilution in RPMI medium 1:100, Millipore, Burlington, MA, USA, 05-369, epitope—intracellular part of α1-subunit) for an hour in a CO_2_-incubator. After the washout, the cells were stained with secondary anti-mouse IgG conjugated to FITC (Santa Cruz Biotechnology, Dallas, TX, USA, sc-2010) and anti-rabbit IgG conjugated to Texas Red (Santa Cruz Biotechnology, Dallas, TX, USA, sc-2780), and incubated for 30 min in a CO_2_-incubator. Before the imaging, nuclei were stained with NucBlue (Invitrogen, ThermoFisher Scientific, MA, USA, R37605).

#### 2.4.3. Laser Scanning Confocal Microscopy 

The attached cells in the glass-based Petri dishes were covered with PBS with 1 mM Ca^2+^/0.5 mM Mg^2+^ and imaged using a confocal microscope Leica TCS SP5 (Leica, Wetzlar, Germany). Na,K-ATPase was labeled with FITC-conjugated antibodies and imaged using a 488 nm argon laser. Aβ_42_ was labeled with Texas Red-conjugated antibodies and imaged using a 594 nm HeNe laser. The resulting images were analyzed using LAS X imaging software (Leica, Wetzlar, Germany). 

### 2.5. Co-Localization Studies with Proximity Ligation Assay

In Situ Proximity Ligation Assay was performed with a Duolink kit (Sigma-Aldrich, St. Louis, MO, USA) according to the Duolink^®^ PLA Fluorescence Protocol (https://www.sigmaaldrich.com/technical-documents/protocols/biology/duolink-fluorescence-user-manual.html; accessed on 14 February 2020) with minor adjustments for primary immunolabeling, which are described below. PLA detects sites where the studied molecules are located no further than 40 nm from each other, making it possible to determine protein-protein interactions with sensitivity at the level of single molecules [31,32].

#### 2.5.1. Primary Immunolabeling of Aβ_42_ and Na,K-ATPase

To study the interaction of Aβ and Na,K-ATPase, SH-SY5Y cells were washed with serum-free RPMI and incubated with 100 nM or 10 µM of Aβ_42_ in serum-free RPMI for 1 h at 37 °C in CO_2_-incubator. After the treatment with Aβ_42_, the cells were washed twice with serum-free RPMI. Primary immunolabeling was performed by different protocols, depending on the antigen used for detection. Both anti-Aβ_42_ and anti-β1-Na,K-ATPase staining was performed on live cells prior to fixation/permeabilization. The cells were incubated with a mixture of anti-Aβ_42_ rabbit monoclonal (1:50, Sigma-Aldrich, St. Louis, MO, USA, A3356) and anti-β1-Na,K-ATPase (1:100, Millipore, Burlington, MA, USA, 05-382) mouse monoclonal antibodies in serum-free RPMI for 30 min at 37 °C in a CO_2_ incubator.

Then, the cells were washed twice with PBS containing 1 mM Ca^2+^ and 0.5 mM Mg^2+^ (Ca^2+^/Mg^2+^ PBS), and fixed in 4% paraformaldehyde at room temperature for 10 min. After the fixation, the cells were washed thrice with ice-cold Ca^2+^/Mg^2+^ PBS and blocked in Duolink blocking solution, according to the manufacturer’s protocol.

For Aβ_42_/α1-Na,K-ATPase staining, cells were incubated with anti-Aβ_42_ 22−35 rabbit monoclonal antibodies (1:50, Sigma-Aldrich, St. Louis, MO, USA, A3356) in serum-free RPMI for half an hour at 37 °C in a humidified chamber. The cells were washed twice with Ca^2+^/Mg^2+^ PBS and fixed in 4% paraformaldehyde at room temperature for 10 min. After the fixation, cells were washed thrice with ice-cold Ca^2+^/Mg^2+^ PBS and permeabilized for 10 min in 0.5% Saponin (Sigma-Aldrich, St. Louis, MO, USA) in Ca/Mg PBS at room temperature. Cells were again washed thrice with Ca^2+^/Mg^2+^ PBS and blocked with Duolink blocking solution according to the manufacturer’s protocol. After the blocking, the cells were incubated with anti-α1-Na,K-ATPase mouse monoclonal antibodies (1:100, Millipore, Burlington, MA, USA, 05-369) in Duolink Antibody diluent for half an hour at 37 °C in a humidified chamber in a CO_2_ incubator.

To control the specificity of the results, PLA was also performed in the absence of either anti-Aβ_42_ antibody, anti-Na,K-ATPase antibodies, or both primary antibodies (Supplementary Figure S2).

After the primary immunolabeling, a PLA assay was performed according to the manufacturer’s protocol, using 40 µL reaction volume for the 35 mm glass-based Petri dish. In the cells not treated with primary antibodies or treated with only one antibody, as well as in the cells not treated with Aβ_42_, the PLA signal was either absent or manifested as uncharacteristic large spots (Supplementary Figure S2).

#### 2.5.2. Primary Immunolabeling of Src Kinase and Na,K-ATPase

SH-SY5Y cells were fixed and permeabilized as above and blocked with Duolink blocking solution, according to the manufacturer’s protocol. After the blocking, the cells were incubated with a mixture of 1:100 anti-Src rabbit monoclonal antibodies (Cell Signaling, Danvers, MA, USA, 2108S) and 1:100 anti-α1-Na,K-ATPase mouse monoclonal antibodies (Millipore, Burlington, MA, USA, 05-369) diluted in Duolink Antibody diluent for 30 min at 37 °C in a CO_2_-incubator. In the cells not treated with primary antibodies or treated with only one antibody, PLA signal was absent (Supplementary Figure S2).

#### 2.5.3. Fluorescent and Confocal Imaging for Proximity Ligation Assay 

After the final wash (see Duolink^®^ PLA Fluorescence Protocol), 100 µL Ca^2+^/Mg^2+^ PBS was added to the cells. To label the cell bodies, washed cells were incubated with 500 nM SYTO RNASelect Green (Invitrogen, ThermoFisher Scientific, MA, USA, S32703) in Ca^2+^/Mg^2+^ PBS for 20 min at room temperature, and then washed twice with Ca^2+^/Mg^2+^ PBS for 5 min each. To stain the nuclei, one drop of Hoechst 33342 Ready Flow Reagent (Invitrogen, ThermoFisher Scientific, MA, USA, R37165) was added to each dish and incubated for 5 min without washing. After the staining, the cells were imaged in Ca^2+^/Mg^2+^ PBS using a confocal Leica TCS SP5 laser-scanning microscope (Leica, Wetzlar, Germany) equipped with an HCX PLAPO CS 63Ч1.4 oil immersion lens. The image acquisition parameters were as follows:

- Hoechst fluorescence (DNA staining) with excitation at 405 nm and emission at 414–487 nm;
- RNASelect Green fluorescence, excitation at 488 nm, emission at 495–590 nm;
- Duolink Detection Reagent (Red) fluorescence, excitation at 594 nm, emission at 600–652 nm.

Images were processed using LAS X software (Leica, Wetzlar, Germany).

### 2.6. Assessment of the Level of Na,K-ATPase and Redox Status of the Cells

SH-SY5Y cells were grown in 6- or 12-well plates. SH-SY5Y cells were washed with serum-free RPMI and then incubated with 100 nM Aβ_42_, or an equivalent amount of DMSO, for 30 or 60 min in serum-free medium. Then, the cells were washed with serum-free RPMI medium and incubated with antibodies for β1-isoform of Na,K-ATPase (1:100, Millipore, Burlington, MA, USA, 05-382) for an hour in a CO_2_-incubator at 37 °C. After washing off the primary antibodies, the cells were incubated with a secondary antibody (anti-mouse IgG) conjugated to FITC (Santa Cruz Biotechnology, Dallas, TX, USA sc-2010) for 30 min in a CO_2_-incubator. Analysis of the cells was performed using a flow cytometer BD LSR Fortessa (Becton Dickinson, Franklin Lakes, NJ, USA).

To assess the level of reduced glutathione and reactive oxygen species (ROS), cells were harvested, stained with ROS and thiol-specific fluorescent dyes, washed and incubated with 100 nM Aβ_42_ for 10 or 30 min. The ROS level was assessed using the dihydrorhodamine 123 (DHR) dye (Ex/Em = 488/525 nm; Invitrogen, ThermoFisher Scientific, MA, USA, D23806). Assessment of the level of reduced glutathione (GSH) was performed using the dye ThiolTracker Violet (Ex/Em = 405/526 nm; Invitrogen, ThermoFisher Scientific, MA, USA T10095). These parameters were recorded for the cells with intact membrane. Cells with damaged membranes were detected by staining with propidium iodide (PI; Sigma-Aldrich, St. Louis, MO, P4170) (Ex/Em = 535/617 nm). A Cellular Redox sensor (ThermoFisher Scientific, MA, USA, P36243) was used to estimate the oxidized glutathione/reduced glutathione ratio (GSSG/GSH). For this, cells were transfected with a baculovirus vector in accordance with the manufacturer’s protocol and then incubated for 10 and 30 min with 100 nM Aβ_42_, as described above. The change in the GSSG/GSH ratio was determined by calculating the ratio of the fluorescence intensity values at a wavelength of 535 nm, obtained with excitation at the wavelengths of 488 and 405 nm, at the initial moment of time and at a given incubation time. Analysis of the cells was performed using a flow cytometer BD LSR Fortessa (Becton Dickinson, Franklin Lakes, NJ, USA).

### 2.7. Analysis of Src Kinase Phosphorylation Levels in SH-SY5Y Cells

SH-SY5Y cells were grown in 6- or 12-well plates. SH-SY5Y cells were exposed to 100 nM, 500 nM and 2 µM of Aβ_42_ for 30 min in serum-free RPMI-1640 at 37 °C with 5% CO_2_ in a CO_2_ incubator. In the experiments with pNaktide, cells were preincubated with 1 mM of pNaKtide (LifeTein) before the addition of Aβ_42_ for 1 h in serum-free RPMI-1640 at 37 °C with 5% CO_2_ in a CO_2_ incubator. For the assessment of hypoxia effect, cells were grown for 24 h in a hypoxic chamber (37 °C, 1% O_2_; 5% CO_2_, 94% N_2_, Whitley H45 HEPA Hypoxistation) prior to the Aβ_42_ application at hypoxic conditions. Incubation of cells with Aβ also was carried out in the Hypoxistation. Control cells were grown in a CO_2_ incubator (37 °C, 20% O_2_; 5% CO_2_). Then, SH-SY5Y cells were incubated with Aβ_42_ for 30 min and after that lysed in the RIPA-buffer (25 mM tris-HCl, pH 7.6, 150 mM NaCl, 1% Nonidet-P40, 0.1% SDS, 1% sodium deoxycholate) containing the protease inhibitors cocktail (Roche, 11836145001), phosphatase inhibitors cocktail (Roche, 4906837001), 5 µM thiorphan (Cayman Chemical, Ann Arbor, MI, USA, 15600) with stirring at 4 °C for 1 h. The lysates were then centrifuged at 16,100× *g* for 10 min at 4 °C and the supernatant was collected.

The cell lysates were separated on 10% SDS PAGE electrophoresis and transferred to a PVDF-membrane (Bio-Rad, Hercules, CA, USA, 1620137). The membrane was blocked in 5% nonfat milk in TBST (50 mM Tis-HCl, pH 7.4, 150 mM NaCl, 0.1% Tween-20), and incubated with primary rabbit antibodies to phospho (Tyr 416)-Src kinase (p-Src; Cell Signaling Technology, Danvers, MA, USA, 6943S) or total Src kinase (Cell Signaling Technology, Danvers, MA, USA, 2108S) in TBST overnight at +4 °C. Then, the membrane was incubated with HRP-conjugated secondary antibodies (ThermoFisher Scientific, MA, USA, A16035) and imaged with chemiluminescence SuperSignal™ West Femto Maximum Sensitivity Substrate kit (ThermoFisher Scientific, MA, USA, 34096) using Bio-Rad ChemiDoc MP instrument (Bio-Rad, Hercules, CA, USA). Densitometric analysis was performed with Image Lab 6.0.1 program (Bio-Rad, Hercules, CA, USA) and the results were expressed as ratio of phospho-Src to total Src band intensity (phospho-Src/Src).

### 2.8. Phosphorylation of Src Kinase In Vitro

Na,K-ATPase (3 µg) purified from duck salt glands, isolated as described earlier [15], was incubated with 10 µM of Aβ_42_, 10 µM of reverse peptide 42−1 (CP_Aβ_) or 1 mM of ouabain at room temperature for 30 min in 36 µL PBS containing 5 mM MgCl_2_. Then, 25 ng (in 1 µL of storage buffer) of Src kinase (Abcam, Cambridge, UK, 79635), which were mixed and further incubated for 5 min. The phosphorylation reaction was started by the addition of 3 mM ATP; the total volume of the reaction mixture was 40 µL. After 15 min incubation at 37 °C, the reaction was stopped by adding 40 µL of 2× Sample Buffer (Novex, ThermoFisher Scientific, MA, USA, LC2676) containing 5% β-mercaptoethanol, and heated for 5 min at 80 °C. Samples were analyzed by Western blot using antibodies to phospho(Tyr416)-Src and total Src as described above.

### 2.9. Microscale Thermophoresis

The capacity of Na,K-ATPase to bind the Src kinase was measured with the Microscale Thermophoresis (MST) [33]. Src kinase (Abcam, Cambridge, UK, 79635) was labeled via His-tag with the Monolith His-Tag Labeling Kit RED-tris-NTA second Generation kit, according to the manufacturer’s protocol. The experiments were carried out in a 50 mM Tris/HCl buffer (pH 7.4) containing 150 mM NaCl, 10 mM MgCl_2_. Serial two-fold dilutions of the unlabeled Na,K-ATPase with 50 mM Tris/HCl buffer (pH 7.4) containing 150 mM NaCl, 10 mM MgCl_2_ were conducted to obtain a set of samples with Na,K-ATPase concentrations ranging from 0.2 nM to 16 μM. RED-tris-NTA-labeled Src kinase concentration was constant (50 nM).

Samples were loaded into Monolith NT.115 Premium Capillaries and MST analysis was performed using the Monolith NT.115 system (Nano Temper Technologies GmbH, München, Germany). The LED/excitation power was 100%, the MST power was 40%. Data analysis was performed using MO.Affinity Analysis software v.2.3 (Nano Temper Technologies GmbH, München, Germany).

### 2.10. Measurement of Na,K-ATPase Transport Activity by Atomic Adsorption Spectrometry

Activity of the Na,K-ATPase was measured as the ouabain-sensitive component of the Rb^+^ influx rate (Rb^+^ was used as a specific analog of K^+^). SH-SY5Y cells in 6-well plates were exposed to 100 nM Aβ_42_ or 100 µM ouabain for 10 min in RPMI-1640 with 5% CO_2_ at 37 °C. After 10 min, RbCl was added to the final concentration of 2.5 mM, and the cells were incubated for 20 min at the same conditions. To identify the Rb^+^ content the cells were transferred onto ice, experimental medium was removed, and cells were washed 3 times with 3 mL of an ice-cold 0.1 M MgCl_2_. Then, 1 mL of trichloroacetic acid (TCA) was added to each well and samples were incubated at 4 °C overnight for complete extraction of ions from the cells. Lysed cells were scraped and centrifuged for 10 min at 16,100 × *g* at 4 °C. Supernatant was quantified by flame atomic absorption spectrometry using the Kvant-2M1 spectrometer (Cortec, Moscow, Russia) with propane-air mixture in accordance with the manufacturer’s manual. Solutions of RbCl (0.2–4 mg/L Rb^+^) in 5% TCA were used for calibration. Protein precipitates were resuspended in 0.5 mL of 0.1 M NaOH and protein concentrations were measured by bicinchoninic acid (BCA) assay kit (Sigma-Aldrich, St. Louis, MO, BCA1-1KT) according to the manufacturer’s protocol. The Rb^+^ content was normalized to the total protein amount in each sample. Total Rb^+^ uptake is a unidirectional Rb^+^ accumulation in the cells in the absence of ouabain. Passive Rb^+^ uptake is a residual ouabain-independent influx component observed in the cells where Na,K-ATPase was inhibited by ouabain. Active Rb^+^ uptake is an uptake mediated by the Na,K-ATPase, which was calculated as a difference between the total and the residual uptake. The absence of detectable efflux of the label during the first 30 min of incubation was earlier confirmed using ^86^Rb^+^ [15]. Hence, we have expressed the data as the influx of Rb^+^ per hour.

### 2.11. Modeling the Interaction of Human Src Kinase with the Nucleotide Binding Domain of β1-Subunit of Human Na,K-ATPase

The structure of human α1-Na,K-ATPase molecule in the E1P conformation was constructed by modeling point mutations in the *Sus scrofa* Na,K-ATPase (PDB:3WGU solved at 2.8 Ǻ resolution), adding DDPC membrane, and performing subsequent relaxation for 50 ns by molecular dynamics (MD) using GROMACS [34] software. Src kinase structure was taken from PDB (PDB:2SRC). Global fullblind and targeted docking of these structures was carried out with HADDOCK [35] and PatchDock [36] servers using Na,K-ATPase nucleotide binding domain (NBD) (377–588 residues) and kinase domain of Src kinase (residues 267–520). Qasdom server [37] (http://qasdom.eimb.ru/qasdom.html, Moscow, Russia) was used to identify the Na,K-ATPase:Src kinase interactions in the obtained complexes. The first 20 complexes from the PatchDock server, 10 best complexes refined with the FireDock [38] utility, and 40 complexes from HADDOCK were used. As a result, the sum of atomic contacts between Na,K-ATPase and Src kinase over all 70 docking complexes for each residue of Na,K-ATPase and Src kinase was obtained. The two best docking structures from the QASDOM rating were used for the MD simulation for 50 ns. After that, the number of contacts between Na,K-ATPase and Src kinase was calculated and the structure with the highest number of contacts was submitted to MD simulation for further 50 ns. Thus, an equilibrium structure of Na,K-ATPase:Src kinase complex after 100 ns MD was obtained. All initial structures submitted for MD were energy minimized consecutively with the steepest descent and conjugated gradients algorithms and equilibrated in water with the NaCl concentration of 150 mM under position restraints for 1 ns in NVT and NPT ensembles, respectively. The CHARMM36 [39] force field was applied. Simulations were carried out using the particle mesh Ewald technique with repeating boundary conditions and 1 nm cut-offs, utilizing the LINCS constraint algorithm with a 2-fs time step. During the MD simulation the constant temperature of 300 K was maintained for the two temperature coupling groups “Protein” and “Non-Protein”.

### 2.12. Statistical Analysis

All experimental data are shown as mean values ± standard deviations of mean (SD), with the number of independent experiments indicated in Figure legends. The statistical difference between experimental groups was analyzed by one-way ANOVA with Tukey correction for multiple comparisons. Probability values (p) less than 0.05 were considered significant. Statistical analysis was performed using GraphPad Prism 9.1.2 software (GraphPad Software Inc., San Diego, CA, USA).

## 3. Results

### 3.1. Aβ_42_ Co-Localizes with Na,K-ATPase and Initiates Src Signaling

The set of experiments was performed to test if Aβ_42_ could be a ligand for the Na,K-ATPase receptor, triggering Src kinase activation. First of all, the co-localization of the Na,K-ATPase with Aβ_42_ was probed.

**Figure 1:**
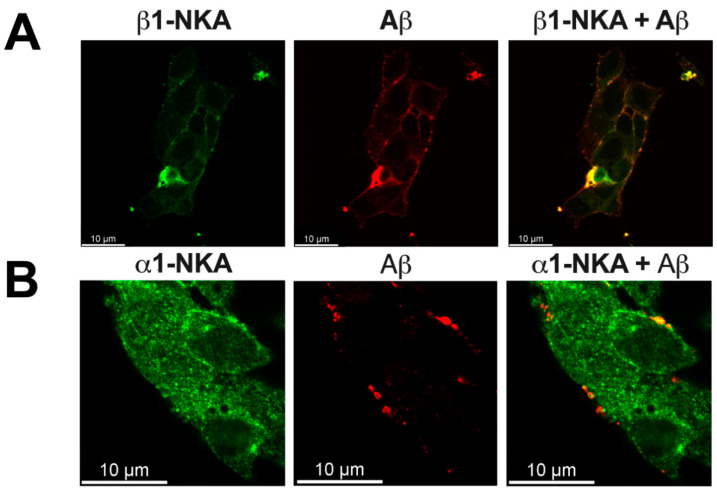

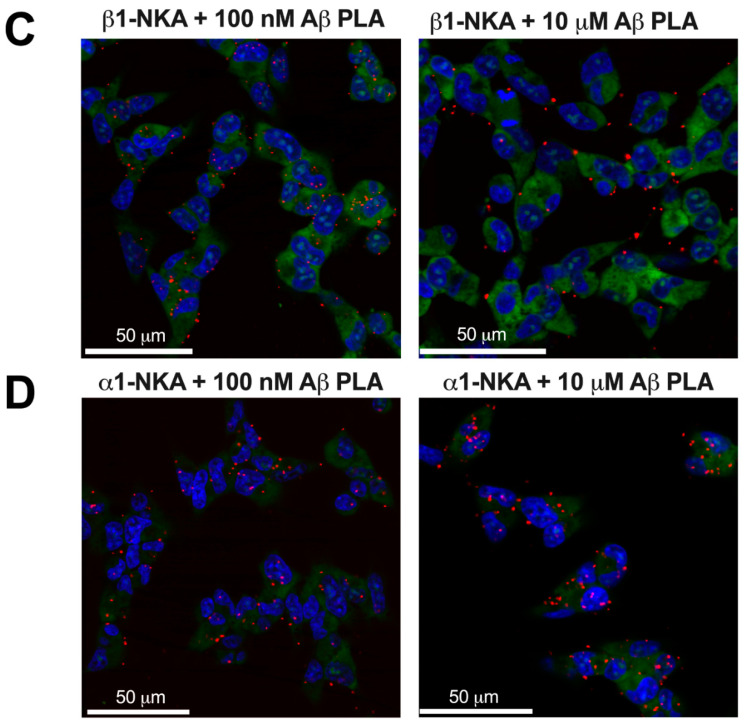
Aβ_42_ co-localizes with Na,K-ATPase. (**A**,**B**) Co-localization studies with confocal microscopy. Representative immunofluorescence images of neuroblastoma cells SH-SY5Y treated with 40 µM Aβ_42_ for 2 h. The distribution of (**A**) β1- and (**B**) α1-subunits of Na,K-ATPase (green fluorescence), the distribution of Aβ_42_ (red fluorescence), and the merged (β1/α1-subunit and Aβ_42_) image. Scale bar—10 µm. (**C**,**D**) Co-localization studies with Proximity Ligation Assay (PLA) in SH-SY5Y neuroblastoma cells. The close proximity sites, where the studied molecules are closer than 40 nm to each other, are visualized as red dots using the Duolink Red detection reagent. The confocal merged images of Hoechst fluorescence (blue), RNASelect (green) and Duolink Red (red) fluorescence are presented. (**C**) Close proximity of Aβ_42_ and Na,K-ATPase β1-subunit in SH-SY5Y cells treated with 100 nM and 10 µM Aβ_42_ for 1 h. Scale bar—50 µm. (**D**) Close proximity of Aβ_42_ and Na,K-ATPase α1-subunit in SH-SY5Y neuroblastoma cells treated with 100 nM and 10 µM Aβ_42_ for 1 h. Scale bar—50 µm.

It was observed by confocal microscopy that when SH-SY5Y neuroblastoma cells were treated with Aβ_42_ it co-localized with both the β1 and α1-subunits of Na,K-ATPase on the surface of cells (Figure 1A,B, Supplementary Figure S1 and Supplementary Movie S1). The Proximity Ligation Assay (PLA) was also used to test co-localization of Aβ and Na,K-ATPase on the cell plasma membrane. PLA detects sites where the studied molecules are located no further than 40 nm from each other, identifying protein-protein interactions with the single-molecule sensitivity [31,32]. It was determined that in SH-SY5Y cells Aβ_42_ co-localizes with both the β1- and α1-subunits of Na,K-ATPase (Figure 1C,D). Co-localization was observed when cells were treated either with 10 μM or 100 nM Aβ_42_. In cells untreated with Aβ_42_ the PLA signal was not detected (Supplementary Figure S2).

**Figure 2:**
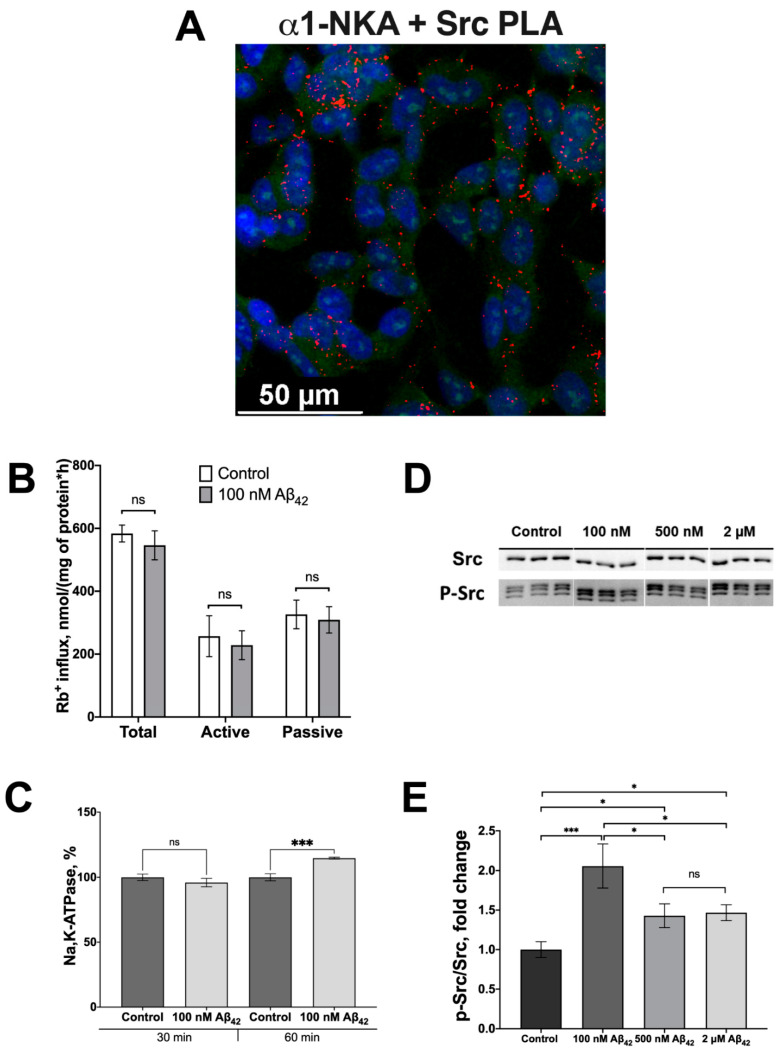
Aβ_42_ activates Src-kinase in nanomolar concentrations. (**A**) Co-localization studies with Proximity Ligation Assay. Close proximity of Na,K-ATPase α1-subunit and Src kinase in the SH-SY5Y neuroblastoma cells. The confocal merged image of Hoechst fluorescence (blue), RNASelect (green) and Duolink Red (red) fluorescence is presented. Scale bar—50 µm. (**B**) The effect of Aβ_42_ on the Na,K-ATPase transport activity in SH-SY5Y cells. K^+^ (Rb^+^) influx after 30 min treatment with 100 nM Aβ_42_. Total Rb^+^ influx into the cells was measured in the absence of ouabain (Total); “Passive” denotes ouabain-resistant component of Rb^+^ influx in the sample where ouabain was added. Difference between the total and the passive fluxes gives the active (Active) Rb^+^ influx mediated by the Na,K-ATPase. (**C**) The changes in Na,K-ATPase levels in SH-SY5Y neuroblastoma cells after 30 or 60 min of incubation with 100 nM Aβ_42_. Na,K-ATPase levels were evaluated by flow cytometry. (**D**,**E**) Dose-dependent activation of Src by Aβ_42_. The ratio of phospho(Tyr)-416 Src to the total Src has been calculated. The phosphorylated and total Src levels have been measured with Western blot in SH-SY5Y neuroblastoma cells treated with 100 nM, 500 nM and 2 µM of Aβ_42_ for 30 min and normalized for control. Mean values ± SD from at least three independent experiments are shown. *—*p* < 0.05, ***—*p* < 0.001 compared to the control, ns—nonsignificant.

Since Src kinase is a downstream messenger for Na,K-ATPase [22,23,24,30,40], an intracellular pool of Src kinase associated with the α1-subunit of Na,K-ATPase is required for the activation of Src kinase triggered by Aβ binding with Na,K-ATPase. Using PLA, co-localization of Src kinase with the α1-subunit of Na,K-ATPase in SH-SY5Y cells was observed (Figure 2A, Supplementary Figure S2). The value of dissociation constant (K_d_) for Na,K-ATPase:Src kinase complex was observed by MicroScale thermophoresis (MST) to be equal to 0.21 ± 0.04 µM (Supplementary Figure S3).

Earlier, it was shown that high concentrations of Aβ_42_ (10 µM) inhibit the transport activity of Na,K-ATPase in SH-SY5Y cells [15]. To exclude the possible effect of changes in the concentration of Na^+^ and K^+^ ions on the activation of Src kinase due to inhibition of Na,K-ATPase by Aβ_42_, the impact of Aβ_42_ at 100 nM was evaluated. Such Aβ concentration is too low to affect active transport of K^+^ and Na^+^ by Na,K-ATPase (Figure 2B). Furthermore, the abundance of Na,K-ATPase at the membrane surface was not affected by the exposure to 100 nM of Aβ_42_ for 30 min, as shown by flow cytometry (Figure 2C). Incubation of cells with 100 nM Aβ_42_ for 60 min caused a 30% increase in the membrane pool of Na,K-ATPase (Figure 2C).

While incubation of cells with 10 μM of Aβ_42_ after 30 min, as we have shown in our previous work [15], led to the activation of Src kinase by 30%, 100 nM of Aβ_42_ applied in this study for the same period of time produced an increase in the activated Src kinase by 100% (Figure 2D,E). As the concentration of Aβ_42_ decreases from micromolar to nanomolar, the activating phosphorylation of Src kinase increases (Figure 2D,E).

### 3.2. Activation of Src Kinase by Aβ_42_ Is Mediated by Na,K-ATPase

**Figure 3:**
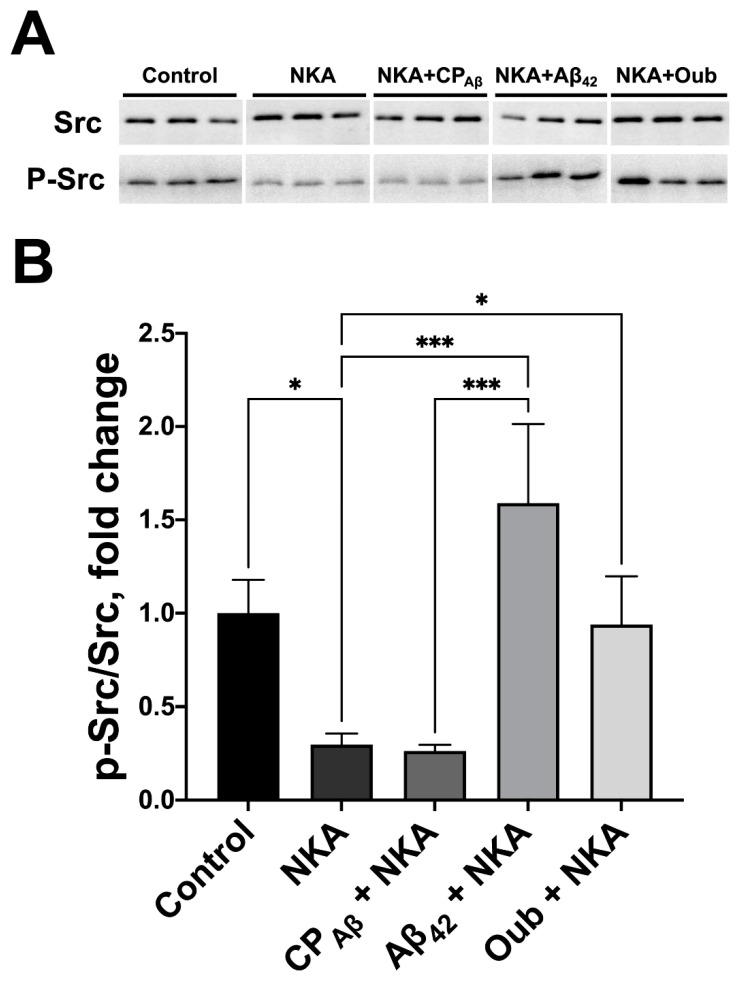
Aβ_42_ activates Src kinase autophosphorylation via Na,K-ATPase *in vitro*. Recombinant human Src kinase was preincubated with Na,K-ATPase (NKA), Aβ_42_, reverse 42−1 peptide (CP_Aβ_) or Ouabain (Oub). Then, samples were incubated with ATP (15 min, 37 °C). (**A**) The p-Src and the total Src levels in the samples were measured with Western blot and (**B**) the p-Src/Src ratio was calculated and normalized for control. Mean values ± SD from at least three independent experiments are shown. *—*p* < 0.05, ***—*p* < 0.001 compared to the control are shown, all other pairwise comparisons were nonsignificant.

Activation of Src kinase was suggested to occur following the dissociation of its kinase domain from the complex with Na,K-ATPase after CTSs binding [24]. Autophosphorylation of Tyr416 residue is essential for the c-Src activation process and it is called activating phosphorylation [41]. Thus, the level of activating phosphorylation was used to evaluate the activity of Src kinase. The effect of Aβ_42_ on activation of Src kinase in complex with Na,K-ATPase was evaluated using the in vitro system for autophosphorylation of Src kinase, comprising purified Na,K-ATPase, Src kinase and ATP. Addition of Na,K-ATPase reduced the Src autophosphorylation at the Tyr416 residue by 70% (Figure 3A,B), which is consistent with the published data [24]. In the presence of Aβ_42_ the activating phosphorylation of Src kinase pre-incubated with Na,K-ATPase was increased to the level of control (Figure 3A,B). Of note, the reverse Aβ peptide 42−1 (CP_Aβ_) did not activate Src kinase in this experimental setting, which suggests the specificity of Aβ-induced Src activation (Figure 3A,B).

**Figure 4:**
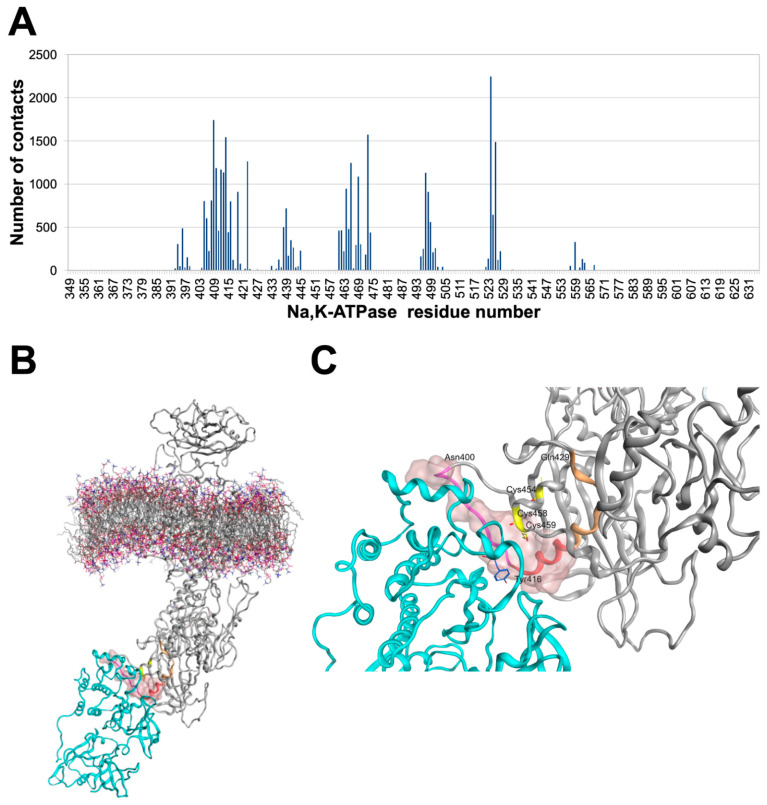
Modeling of Src kinase interaction with Na,K-ATPase. Interaction interface between human Na,K-ATPase and Src kinase studied by molecular modeling. (**A**) Contact frequency histogram of Na,K-ATPase residues according to 70 complexes of Na,K-ATPase:Src kinase obtained by targeted docking on servers PatchDock and Haddock. (**B**) Best rated docking complex after 100 ns of MD. (**C**) Interaction interface between Na,K-ATPase and Src kinase in the best rated docking complex after 100 ns of MD. Src kinase is colored cyan and Na,K-ATPase is colored gray. The interaction surface is shown with translucent pink. Na,K-ATPase residues 400–418 that interact with Src kinase are colored with magenta and red (residue 410–418 are the part of NaKtide peptide sequence). Residues 419–429 of NaKtide peptide sequence that do not participate in interaction with Src kinase are colored beige. Cysteines 454, 458 and 459 that are located inside the interaction are colored yellow. Tyrosine 416 which is located inside the interaction interface is shown.

Structure modeling was employed to determine the amino acid residues involved in the interaction of Src kinase and the human α1-subunit of Na,K-ATPase. A model of the human α1-subunit embedded in the membrane was created based on the existing porcine α1-subunit structure (PDB:3WGU). The kinase domain (267–520 aa) of Src kinase and the nucleotide binding domain (NBD) (377–588 aa) of Na,K-ATPase were used as target sites for the site-directed docking of Src kinase to Na,K-ATPase. Docking results showed that the interaction involves relatively small regions of both proteins, limited by the amino acid residues 400–418, 433–434, 459–468, 492–494, 519–521 in Na,K-ATPase, and 272–280, 348–356, 422, 460–469 in Src kinase. This indicates specificity of the interaction (Figure 4A). The main interaction site of α1-subunit with Src kinase was formed by the residues 400-NQSGVSFDKTSATWLALSR-418. This segment partially overlaps with the region 410–429, corresponding to the sequence of the NaKtide peptide, an inhibitor of the Src kinase:Na,K-ATPase interaction. Residues 419–429 in the modeled structure of Na,K-ATPase were located inside the protein and were inaccessible for interaction (highlighted in Figure 4B,C with beige). Molecular dynamics simulation showed that the resulting complex retains stable structure for 100 ns, with the RMSD of 0.4 nm (Figure 4B,C). Cysteine residues 458 and 459 are shown in Figure 4C with yellow and it is clearly seen that they are located within the interaction interface. According to the model, Tyr416 residue of Src kinase is located in close proximity to the interaction interface on Na,K-ATPase and within the interaction interface of Src-kinase.

**Figure 5:**
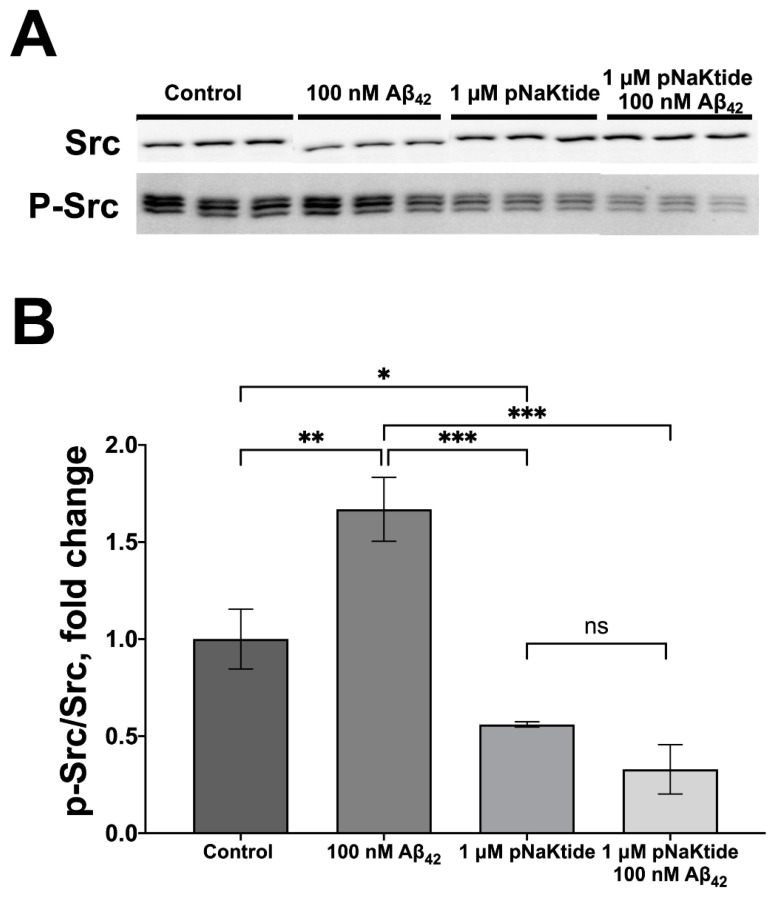
pNaKtide prevents Aβ_42_-induced activation of Src kinase. SH-SY5Y cells were preincubated for 1 h with 1 µM pNaKtide and then the cells were incubated with the medium containing 100 nM Aβ_42_ for 30 min. (**A**) The cells were lysed and the phospho(Tyr416)Src kinase (p-Src) and the total Src levels were measured with Western blot. (**B**) The corresponding p-Src/Src ratio was calculated and normalized for control. Mean values ± SD from at least three independent experiments are shown. *—*p* < 0.05, **—*p* < 0.01, ***—*p* < 0.001 compared to the control, ns—nonsignificant.

A pNaKtide inhibitor formed by the NaKtide peptide (region 410–429 of Na,K-ATPase α1-subunit) linked to the peptide that ensures its penetration into the cell was used to test whether Aβ_42_ activates Src kinase in cells by a mechanism similar to that for CTSs, namely by its release from the complex with Na,K-ATPase. pNaKtide enters the cells and, by binding to Src, prevents its interaction with Na,K-ATPase [30]. Preincubation with pNaKtide prevents an increase in the activating phosphorylation of Src kinase under exposure to 100 nM of Aβ_42_ (Figure 5A,B). Thus, Src activation by Aβ_42_ is not observed if the interaction of Na,K-ATPse with Src kinase is impaired. This confirms that the activation of Src kinase by Aβ_42_ is mediated by Na,K-ATPase. 

### 3.3. Aβ_42_ Affects Cellular Redox-State and Does Not Activate Src Kinase under Hypoxia

**Figure 6:**
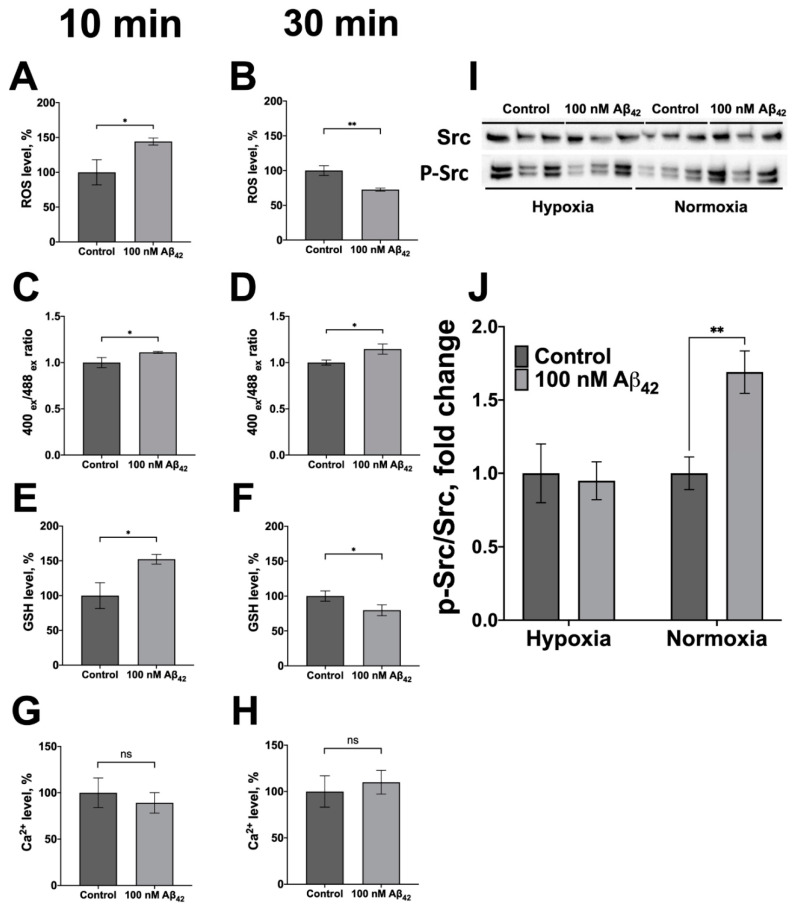
Aβ_42_ alters cellular redox parameters and does not activate Src kinase in hypoxic conditions. (**A**,**B**) The effect of Aβ_42_ on the levels of reactive oxygen species (ROS). (**C**,**D**) The oxidized glutathione/reduced glutathione (GSSG/GSH) ratio. (**E**,**F**) Reduced glutathione (GSH), and (**G**,**H**) Ca^2+^ levels in the SH-SY5Y human neuroblastoma cells. The cells were harvested, stained with fluorescent probes: Dyhydrorhodamine 123 for ROS measurements, ThiolTracker Violet for GSH measurements, and Fluo-4 for Ca^2+^ levels measurements and incubated with 100 nM Aβ_42_ for 10 min (**A**,**C**,**E**,**G**) or for 30 min (**Β**,**D**,**F**,**H**). The GSSG/GSH ratio was determined with Grx1-roGFP genetically encoded ratiometric sensor. The change in the GSSG/GSH ratio was determined by calculating the ratio of the fluorescence intensity values at a wavelength of 535 nm, obtained with excitation at the wavelengths of 488 and 400 nm. All parameters were normalized for control. (**I**,**J**) The ratio of phospho(Tyr-416)-Src to total Src in SH-SY5Y neuroblastoma cells incubated with 100 nM of Aβ_42_ for 30 min under hypoxic conditions (1% O_2_) and standard conditions (20% O_2_) determined with Western blot. (**I**) The representative blot, and (**J**) the corresponding p-Src/Src ratio are presented. Mean values ± SD from at least three independent experiments are shown. *—*p* <0.05, **—*p* <0.01 compared to the control, ns—nonsignificant.

Activation of the receptor function of Na,K-ATPase by CTSs leads to an increase in the reactive oxygen species and a change in the redox status of the cells [42,43,44,45,46], which can substantially affect their functioning. A change in the ROS level, as well as the level of reduced glutathione and the ratio of the oxidized and reduced forms of glutathione in the SH-SY5Y cells after 10 and 30 min of incubation with 100 nM of Aβ_42_ was characterized by flow cytometry. After 10 min of incubation with Aβ_42_, an increase in the ROS level was observed, which after 30 min was reversed to a decrease (Figure 6A,B). Such a short-term increase in ROS is characteristic of the activation of signaling cascades. The oxidized glutathione/reduced glutathione ratio begins to increase after 10 min of incubation, reaching a peak value after 30 min (Figure 6C,D). Accordingly, the reduced glutathione level is decreasing after 30 min of incubation (Figure 6E,F). Consequently, activation of the Src kinase by Aβ_42_, similarly to its activation by CTSs, is accompanied by a change in the redox status of the cells. The level of intracellular Ca^2+^ does not change at the studied time intervals (Figure 6G,H), i.e., intracellular Ca^2+^ is not involved in the observed change in the redox status of the cells.

Ouabain induces intracellular Src kinase activation via Na,K-ATPase [24,25,26,27]. However, in hypoxia Na,K-ATPase-dependent activation of Src by ouabain is not observed due to redox-modification of Na,K-ATPase [47]. We found that under hypoxic conditions (1% O_2_) Aβ_42_ does not activate Src kinase (Figure 6I,J). This observation provides further evidence that activation of Src by Aβ_42_ is mediated by Na,K-ATPase.

## 4. Discussion

Endogenous Aβ_42_ is a critical player in synaptic plasticity and memory within a central nervous system [8]. Its depletion impairs synaptic plasticity and memory, while the addition of exogenous Aβ_42_ precludes these changes. Bulk concentrations of Aβ_42_ in the cerebrospinal fluid (CSF) of healthy subjects are within 0.1–0.2 nM range [48]. However, in the vicinity of synaptic membrane Aβ_42_ concentration may be substantially higher as firstly it is released from the membrane by cleavage of the transmembrane amyloid precursor protein (APP) abundant in synaptic membrane [49] and, secondly, it can be released from intracellular vesicles, where Aβ reaches micromolar concentrations [50]. Thus, the paracrine effect of the newly produced Aβ_42_ could be expected within the upper nanomolar concentration range comparable to the concentrations used in our study. Our data show that the short-term exposure to nanomolar concentrations of monomeric Aβ_42_ on Na,K-ATPase leads to the activation of Src kinase (Figure 2D,E), similar to its activation when CTSs binds to Na,K-ATPase. A shorter Aβ_40_ peptide is more abundant in a healthy brain and CSF [48] and can be important for Na,K-ATPase regulation; however, the interaction with Na,K-ATPase for this proteoform was not studied as Aβ_40_ is less important for accumulation of pathogenic aggregates.

Until now, CTSs have been considered the only ligands which mediate the receptor function of the Na,K-ATPase. The concentration of endogenous CTSs in the plasma of healthy subjects (0.04–0.8 nM for ouabain and 0.2–0.6 nM for marinobufagenin) is close to the concentration of Aβ [51,52]. CTSs are involved in the regulation of the processes related to blood pressure and volume, heart function and other [51] and perform protective and adaptive functions [52,53,54]. High concentrations of CTSs produce a cytotoxic effect due to both inhibition of the activity of Na,K-ATPase and long-term activation of the signaling cascades through Na,K-ATPase [55]. Inhibition of Na,K-ATPase upon prolonged exposure to high concentrations of Aβ_42_ [15,17,18,56] impairs electrotonic properties of neurons and plays an important role in the cytotoxic effect of Aβ. By analogy with CTSs, we suggest that the cytotoxic effects of Aβ_42_ are also associated with the long-term activation of Src kinase through Na,K-ATPase.

Currently, it is believed that pathological processes leading to the impaired functioning of neurons in AD are caused by the oligomeric forms of Aβ [57]. Interaction with oligomeric forms of Aβ_42_ has been shown for Na,K-ATPase with the α3-isoform of the catalytic subunit [17,58]. At the same time, Aβ plays an important role in the regulation of neurogenesis and the formation of memory [4,5,6,7,8]. The physiological function of Aβ_42_ is mediated by its monomeric form [59]. It interacts with Na,K-ATPase containing the α1-isoform of the catalytic subunit, at stoichiometric ratio of 1:1 [15]. Since this isoform is associated with Src kinase [22], the observed rapid activation of Src kinase under exposure to Aβ_42_ indicates that Na,K-ATPase acts as a receptor for Aβ_42_. Unlike the ubiquitous α1-isoform, the α2-isoform of Na,K-ATPase does not interact with Src kinase [22], and the α3-isoform responds to ouabain stimulation by activating extracellular signal-regulated kinase 1/2, but not the Src kinase [60,61]. In addition, the α3-isoform has been shown to bind oligomeric forms of Aβ_42_ [17,58], but not monomers [17].

There are two proposed models describing binding and signal transduction from the α1-subunit of Na,K-ATPase to Src kinase. According to the first one, the α1-subunit and Src kinase form a functional signaling complex [24,27]. Alternatively, it is suggested that Src transiently binds to Na,K-ATPase [62]. Both hypotheses imply the signal transmission by interaction of the α1-subunit with Src kinase. Our data indicate that Na,K-ATPase and Src-kinase form a complex with a K_d_ about 0.2 µM (Supplementary Figure S3). According to the data of Li et al. [30] the SH2-domain of Src kinase is bound to the actuator domain of the α1-subunit in the ATPase:Src kinase signaling complex, and the kinase domain of Src kinase interacts with the NBD domain of the α1-subunit. Binding of CTSs to Na,K-ATPase leads to dissociation of the kinase domain of Src kinase from the complex and activation of the latter as a result of Tyr416 autophosphorylation [30]. Using an in vitro system, we showed that, similarly to CTSs, the interaction of Aβ_42_ with Na,K-ATPase in complex with Src kinase leads to kinase activation (Figure 3A,B).

Using computer simulations, we for the first time predicted and described the specific interaction interface between the nucleotide binding domain of human Na,K-ATPase and the kinase domain of human Src kinase, which is important for the inhibition of Src kinase. In our model Tyr416 of Src kinase was located within the interaction interface with Na,K-ATPase (Figure 4). We suppose that Tyr416 can form stable hydrogen bond with Na,K-ATPase, which hinders Tyr416 phosphorylation and prevents Src activation. This model confirms our experimental data. Specific interaction interface on Na,K-ATPase partially overlaps with the region that was used as a base for creation of Src kinase inhibitor peptide NaKtide [30], which supports the relevance of the model. The interface 400-NQSGVSFDKTSATWLALSR-418 proposed by us could be used to develop new inhibitors of Src/Na,K-ATPase interaction.

Src kinase activation by Aβ_42_ is also observed in neuroblastoma cells (Figure 2D,E). At nanomolar concentrations, that do not inhibit Na,K-ATPase (Figure 2B), Aβ_42_ induces an even higher Src activation (Figure 2D,E) than at micromolar concentrations that inhibit the enzyme [15]. Thus, inhibition of Na,K-ATPase by Aβ_42_ and the resulting change in the intracellular Na^+^/K^+^ ratio analogous to produce by CTSs [44,63], are not required for the induction of Src kinase activation (Figure 2D,E). Activation of Src kinase by Aβ_42_ is impaired by the cell penetrating peptide pNaKtide [30], which prevents interaction of the kinase domain of Src with the Na,K-ATPase NBD domain (Figure 5A,B). α1 subunit was shown to interact with intracellular Src kinase [22], whereas a smaller intracellular domain of β1 subunit is incapable to form such an interaction. In these cells the α1-subunit of Na,K-ATPase also co-localizes with Src kinase (Figure 2A). This confirms that the activation of Src kinase is mediated by the interaction of Aβ_42_ with Na,K-ATPase. The absence of the effect of short-term exposure to 100 nM Aβ_42_ on the level of Na,K-ATPase on the cell surface, and an increase in its level with an increase in incubation time (Figure 2C) is characteristic of the activation of the Na,K-ATPase receptor function. We suggest that it reflects the transport of existing molecules of Na,K-ATPase to the cell surface. Similarly, nanomolar concentrations of CTSs that do not inhibit Na,K-ATPase can both decrease and increase its level in the membrane [64].

Proteins of the Src kinase family, including the Src kinase itself, have a high level of expression in the central nervous system [65,66,67], since they are important enzymes in signaling that support neuronal survival. One of the main consequences of the activation of Src kinase by Aβ_42_ can be a feedback loop regulating the level of Aβ in the synapse. It was shown that phosphorylation of the neuronal trafficking adapter Mint (Munc-18-1 interacting protein) by Src kinase leads to the accumulation of APP in the trans-Golgi network and to a decrease in its transport through neurites to synaptic terminals [68]. On the other hand, Src-dependent activation of phospholipase C leads to an increase in α-secretase activity [69]. An increase in the activity of this enzyme results in a decrease in Aβ production due to greater non-amyloidogenic APP processing. Taken together, these mechanisms should lead to a decrease in the amount of Aβ in the synapse upon the activation of Src kinase by Aβ. Under hypoxic conditions Src-kinase is no longer controlled by Na,K-ATPase, and Aβ_42_ is unable to activate it (Figure 6I,J), while the feedback loop regulating the level of Aβ in the synapse should be disrupted.

**Figure 7:**
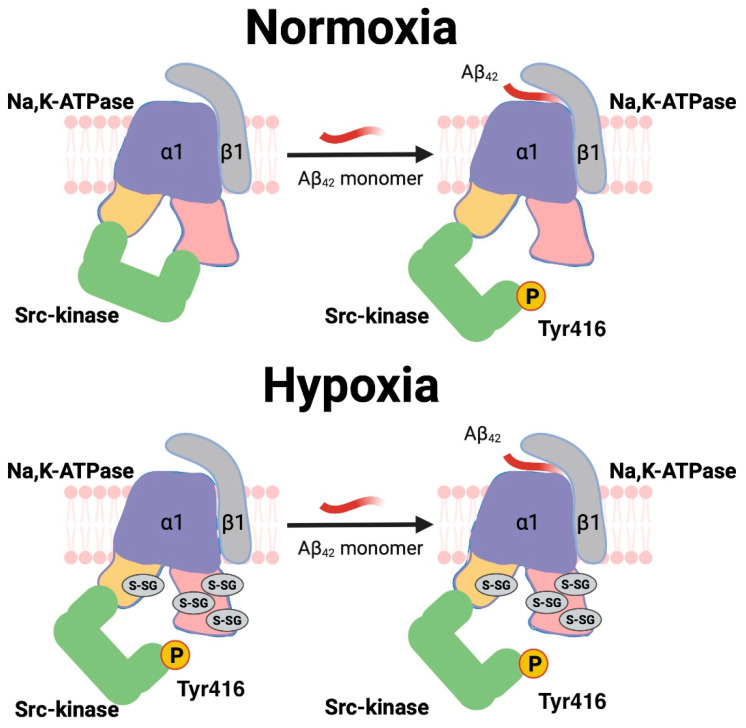
Src kinase regulation mediated by Na,K-ATPase in normoxic and hypoxic conditions. Under normixic conditions, Aβ binding leads to the release of the kinase domain of Src from the complex with the nucleotide binding domain (NBD) of Na,K-ATPase. The release induces Src kinase autophosphorylation at Tyr416 (located in the interaction interface), leading to an increase in the activity. In hypoxia, glutathionylation of the cysteine residues of the Na,K-ATPase NBD domain [73] from the interaction interface (Figure 5) was demonstrated and led to disruption of the interaction between Src kinase and Na,K-ATPase [47]. As a result, the binding of Aβ under hypoxic conditions does not lead to the activation of Src kinase.

Hypoxia is one of the factors that increase risk of AD [70,71]. Intermittent hypoxic treatment reduces the accumulation of Aβ in the brain of model mice with AD and improves their memory [72]. The role of Aβ in the damage and adaptation of cells under hypoxic conditions has not yet been established [71] and the impairment of Src kinase activation by Aβ under the hypoxic conditions that we observed could represent an important element of such regulation. We found that the incubation of cells with Aβ_42_ under hypoxic conditions does not lead to activation of Src kinase in the cells (Figure 6I,J). A similar effect was observed previously for CTSs [47]. The reason for this is the glutathionylation of Na,K-ATPase at the Cys residues 458, 459, induced during hypoxia [73], which leads to disruption of the interface between Src kinase and Na,K-ATPase [47]. As a result, Na,K-ATPase under hypoxic conditions loses control of Src kinase and its activity increases. This result is important for understanding the effect of Aβ_42_ on cells under conditions of ischemia/hypoxia, as it demonstrates that under these conditions the normal signaling function of Aβ_42_ is impaired following the release of Src kinase from the control of Na,K-ATPase. Our findings and suggestions about Src kinase regulation mediated by Na,K-ATPase in normoxic and hypoxic conditions are presented in the Figure 7.

The similar effects of Aβ_42_ and ouabain on Na,K-ATPase could be the result of closely located or overlapping binding sites. However, our recent in vitro data suggest that this is not the case. Aβ_42_ and ouabain do not compete for binding to Na,K-ATPase and do not change each other’s affinity to the enzyme. Molecular modeling is consistent with in vitro studies and shows different binding sites for CTSs and Aβ_42_ at Na,K-ATPase [74].

Aβ_42_ induces a Ca^2+^-independent change in intracellular ROS (Figure 6A,B,G,H). A similar change in ROS was observed in Src-dependent signaling mediated by CTS [43,75,76,77]. In addition, Aβ_42_ leads to an acute change in the thiol redox status of cells accompanied by a decrease in GSH and increase in GSSG/GSH ratio (Figure 6C–F). The change in the redox status induces redox-dependent modifications of proteins, which play an important role in the change of the state of cells affected by Aβ [78,79,80,81]. In particular, incubation of SH-SY5Y cells with Aβ_42_ for 24 h leads to an increase in glutathionylation of Na,K-ATPase [79]. A decrease in the GSH level caused by applying Aβ_42_ was found in vitro and in vivo at long incubation times (24–48 h) [82,83,84]. The rapid change in the redox status, after 10–30 min incubation with Aβ_42_, shown in this study means that the emergence of post-translational protein modifications in the cells under the effect of Aβ should occur much faster than previously assumed [80]. We suggest that the change in the redox status caused by Aβ_42_ due to the induction of glutathionylation of Na,K-ATPase, will disrupt the activation of Src kinase by Aβ_42_, which could constitute a part of the feedback loop.

## 5. Conclusions

The obtained results indicate that the α1-subunit-containing isozyme of Na,K-ATPase is a receptor for Aβ_42_, and their interaction leads to the activation of Src kinase bound to the enzyme. Even at nanomolar concentration, Aβ_42_ rapidly induces the activation of Src kinase and shifts the redox state of the cells. The functioning of this receptor system is impaired during hypoxia. Thus, activation of Src kinase mediated by Aβ_42_ interaction with Na,K-ATPase could be a part of the adaptive, protective and physiologically relevant signaling mechanisms in the brain.

## Data Availability

Not applicable.

## Supplementary Materials

The following supporting information can be downloaded at: https://www.mdpi.com/article/10.3390/cells11172753/s1, Figure S1. The studies of Aβ co-localization with Na,K-ATPase by confocal microscopy. Figure S2. Close proximity of Na,K-ATPase α1-subunit and Src kinase in SH-SY5Y neuroblastoma cells. Figure S3. MTS assay on binding of Src kinase with Na,K-ATPase. Movie S1. The immunofluorescent staining of Aβ42 and Na,K-ATPase in Aβ42-treated SH-SY5Y neuroblastoma cells. 3D reconstruction of confocal microscopy stacks of 10 images with total thickness of 3.4 µm.
