# Supplementary material for: Na,K-ATPase Acts as a Beta-Amyloid Receptor Triggering Src Kinase Activation
Source: Cells. 2022 Sep 3;11(17):2753. doi: 10.3390/cells11172753 (PMC9455167; doi:10.3390/cells11172753)
Supplement: Supplementary file 1 [file cells-11-02753-s001.zip › Supplementary file.pdf]

Supplementary data

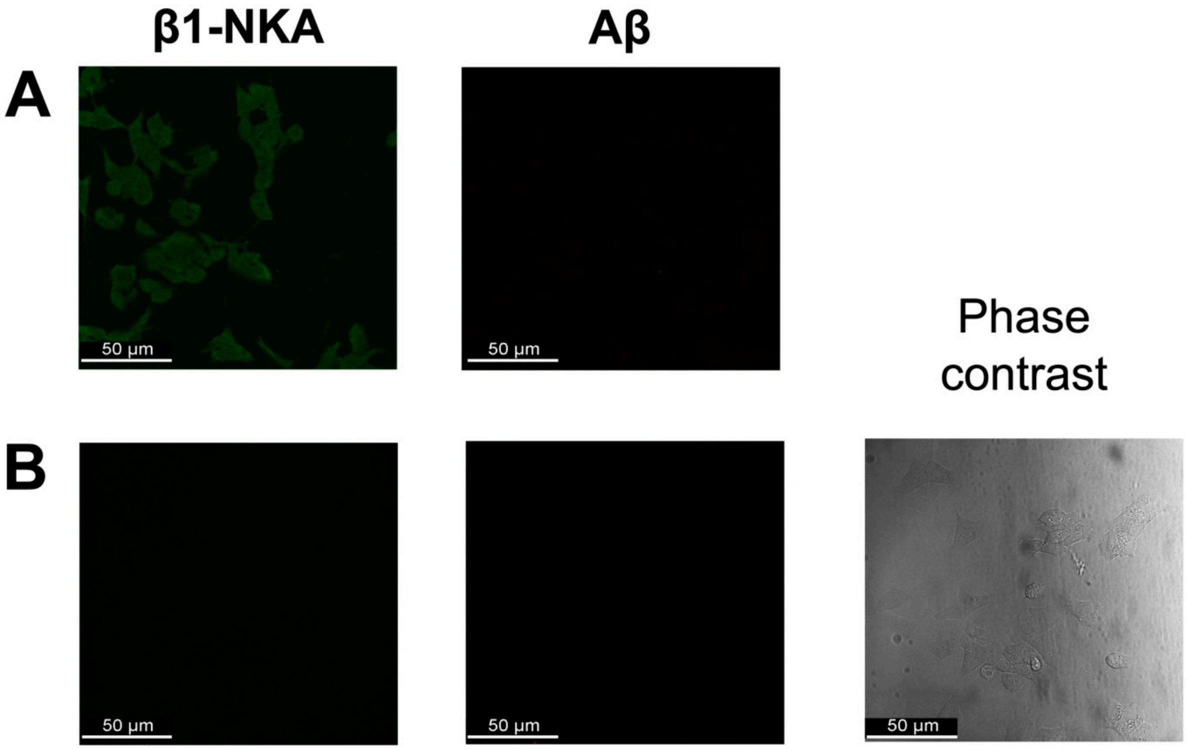

Supplementary Figure S1. The studies of Aβ co-localization with Na,K-ATPase by confocal microscopy.

(A) The control for specificity of primary antibodies. The SH-SY5Y cells were stained with anti-β1-NKA and anti-Aβ antibodies without $A\beta_{42}$ treatment. The distribution of β1-subunits of Na,K-ATPase (left image, green fluorescence). The red fluorescence corresponding to the distribution of anti-$A\beta_{42}$ antibody is absent (right image). Scale bar is shown in the bottom left corner of each image – 50 µm.

(B) The control for specificity of secondary antibodies. SH-SY5Y cells were treated with 40 µM $A\beta_{42}$ for 2 hours and stained with anti-β1-NKA and anti-Aβ antibodies without the secondary (fluorescent) antibodies. The green fluorescence corresponding to distribution of β1-subunits of Na,K-ATPase is absent. The red fluorescence corresponding to distribution of $A\beta_{42}$ is absent. The phase contrast image of cells is shown. Scale bar is shown in the bottom left corner of each image – 50 µm.

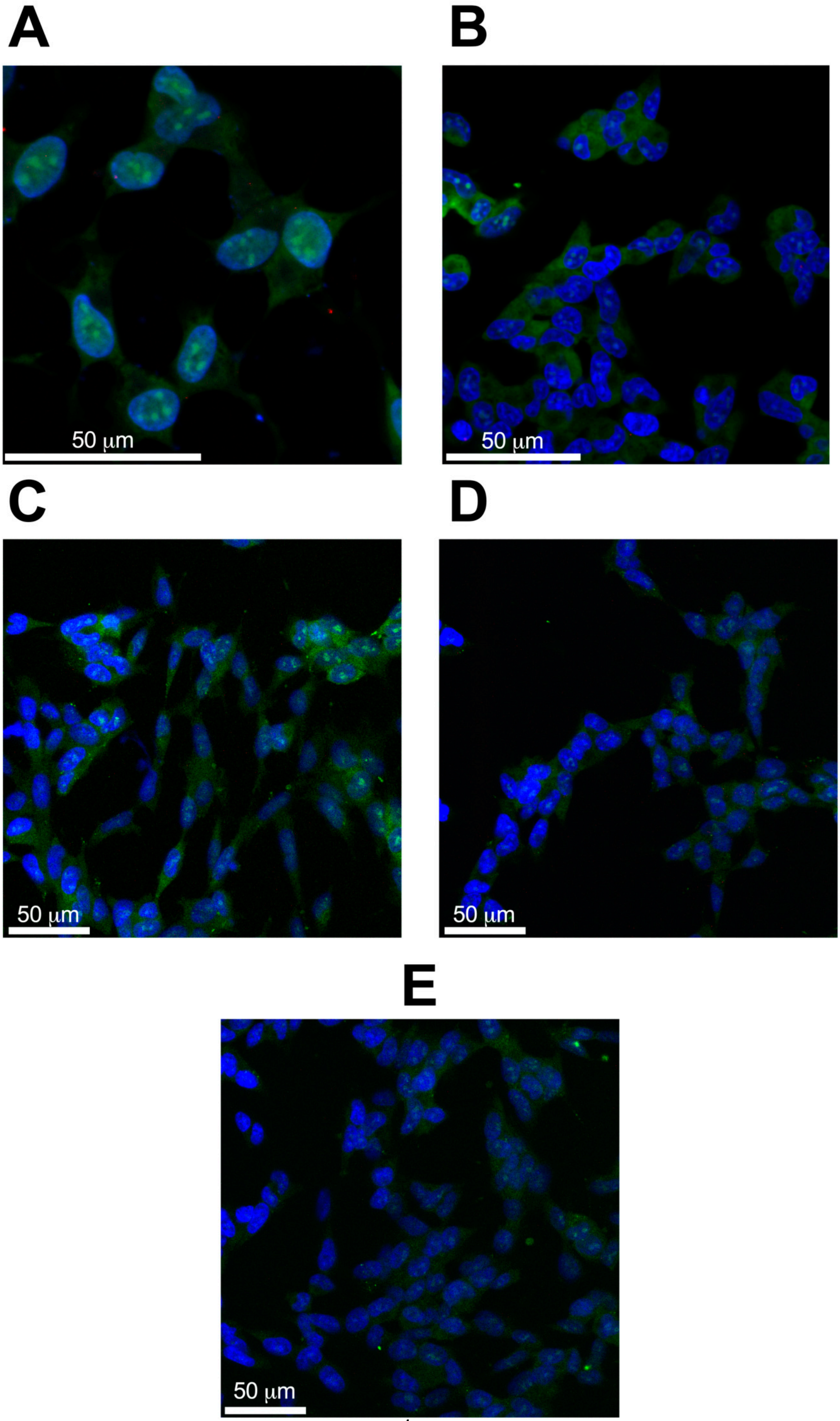
A
B
C
D
E
50 µm
50 µm
50 µm
50 µm
50 µm

Supplementary Figure S2. Close proximity of Na,K-ATPase α1-subunit and Src kinase in SH-SY5Y neuroblastoma cells.

(**A**) SH-SY5Y cells were not treated with $A\beta_{42}$. Both anti-Na,K-ATPase α1-subunit and anti-$A\beta_{42}$ primary antibodies were added. PLA signal is absent.

(**B**) SH-SY5Y cells were not treated with $A\beta_{42}$. Both anti-Na,K-ATPase β1-subunit and anti-$A\beta_{42}$ primary antibodies were added. PLA signal is absent.

(C) SH-SY5Y cells were incubated with only anti-Na,K-ATPase α1-subunit primary antibodies. PLA signal is absent.

(**D**) SH-SY5Y cells were incubated with only anti-Src primary antibodies. PLA signal is absent.

(**E**) SH-SY5Y cells were incubated without primary antibodies. PLA signal is absent.

Merged confocal images showing Hoechst fluorescence (blue), RNASelect (green) are presented. Scale bar is shown the in bottom left corner of each image – 50 µm.

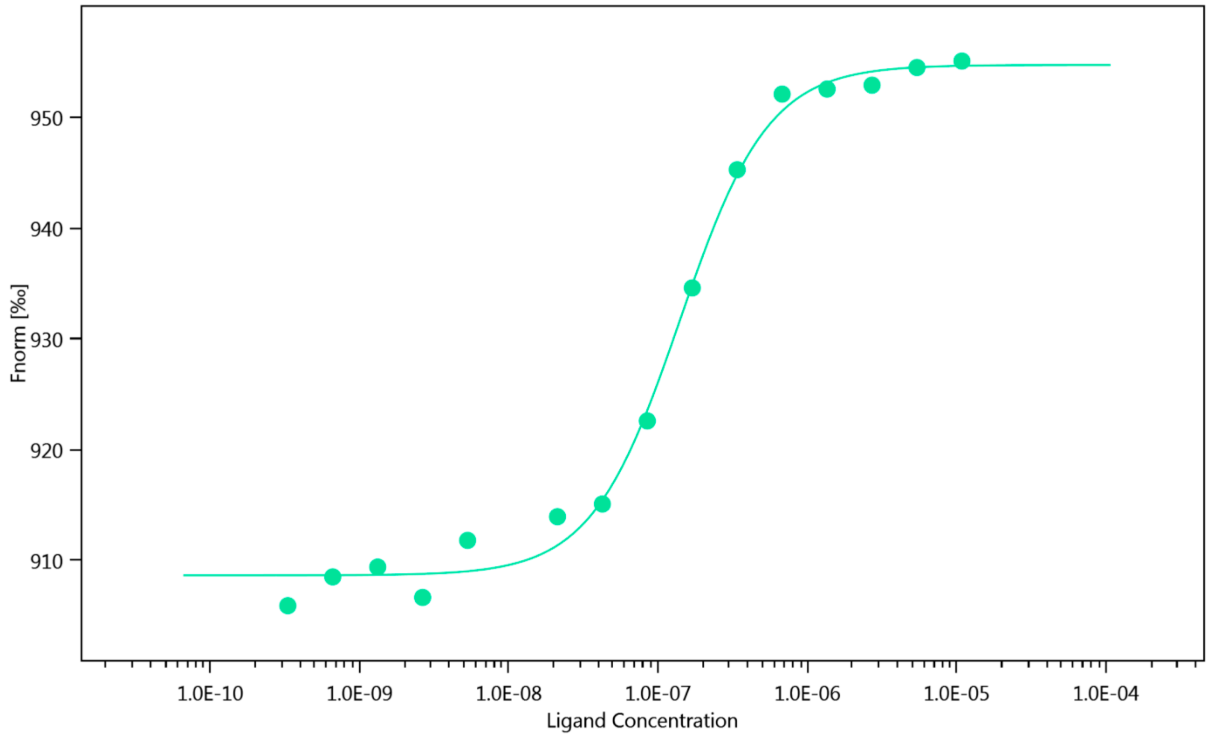

Supplementary Figure S3. MTS assay on binding of Src kinase with Na,K-ATPase.

The Na,K-ATPase was titrated against fluorescent labeled 50 nM Src kinase. The normalized thermophoresis value Fnorm% was plotted against the Na,K-ATPase concentration. Data of one from three independent measurements are shown. Data from all three experiments were used to calculate K_d (see text).

Supplementary Movie S1. The immunofluorescent staining of Aβ_{42} and Na,K-ATPase in Aβ_{42}-treated SH-SY5Y neuroblastoma cells. 3D reconstruction of confocal microscopy stacks of 10 images with total thickness of 3.4 μm.

(A) The distribution of β1-subunits of Na,K-ATPase (green fluorescence).

(B) The distribution of Aβ_{42} (red fluorescence).

(C) The merged visualization of β1-subunits of Na,K-ATPase and Aβ_{42} distributions.

SH-SY5Y cells were treated with 40 μM Aβ_{42} for 2 hours.
